# Clasificación de la expresión del receptor 2 del factor de crecimiento epidérmico humano en tejido mamario canceroso mediante inteligencia artificial

**DOI:** 10.7705/biomedica.7899

**Published:** 2025-12-10

**Authors:** Leidy Verónica Villota, Jessica Julieth Lasso, Elvia Noélia Muñoz, Rubiel Vargas

**Affiliations:** 1 Grupo de Investigación en Sistemas Dinámicos, Instrumentación y Control, Universidad del Cauca, Popayán, Colombia Universidad del Cauca Grupo de Investigación en Sistemas Dinámicos, Instrumentación y Control Universidad del Cauca Popayán Colombia; 2 Unidad de Diagnóstico en Patología SAS, Popayán, Colombia Unidad de Diagnóstico en Patología SAS Popayán Colombia

**Keywords:** neoplasias de la mama, inmunohistoquímica, inteligencia artificial, Breast cancer, immunohistochemistry, artificial intelligence

## Abstract

**Introducción.:**

El análisis histológico y molecular del tejido mamario es clave para el diagnóstico, el pronóstico y el tratamiento del cáncer de mama. Entre los biomarcadores evaluados, se destacan los receptores de progesterona, los de estrógeno y el receptor 2 del factor de crecimiento epidérmico humano (HER2). La sobreexpresión de HER2 indica un subtipo agresivo de cáncer de mama, aunque permite el uso de terapias dirigidas que mejoran la tasa de supervivencia. No obstante, su evaluación enfrenta desafíos, desde la calidad de las muestras hasta la variabilidad en la interpretación. El *College of American Pathologists* clasifica la sobreexpresión de HER2 en cuatro categorías, pero la variabilidad en la expresión cercana al 10 % puede generar confusión.

**Objetivo.:**

Presentar una técnica basada en la inteligencia artificial para clasificar células con sobreexpresión de HER2 en las placas histológicas.

**Materiales y métodos.:**

Se aplicó la metodología *Cross-Industry Standard Process for Data Mining* (CRISP-DM) en muestras de 89 pacientes de la Unidad de Diagnóstico en Patología, abarcando los cuatro niveles de HER2. Se utilizaron redes neuronales y modelos de *Vision Transformer* (ViT) afinados mediante transferencia de aprendizaje. Además, se evaluó la facilidad de uso y, finalmente, la eficiencia del *software* presentado.

**Resultados.:**

Con el modelo ViT-B/16, se obtuvo una exactitud del 90,65 % en la clasificación, mientras que la herramienta evaluada generó un grado aceptable de satisfacción con su aplicación clínica.

**Conclusión.:**

La inteligencia artificial demostró gran precisión y concordancia en la clasificación del HER2, redujo la variabilidad diagnóstica y mejoró la objetividad, aunque aún se requiere optimizar la eficiencia del procesamiento.

## Abstract (en)

**Introduction.:**

Histological and molecular analysis of breast tissue is essential for the diagnosis, prognosis, and treatment of breast cancer. Key biomarkers include progesterone and estrogen receptors, as well as the human epidermal growth factor receptor 2 (HER2). HER2 overexpression indicates an aggressive subtype of breast cancer but enables targeted therapies that improve survival rates. However, its evaluation faces challenges, ranging from sample quality to interpretation variability. The College of American Pathologists classifies HER2 overexpression into four categories, but variations around the 10% expression threshold can lead to misinterpretations.

**Objective.:**

To present an automated technique for classifying HER2-overexpressing cells in histological slides.

**Materials and methods.:**

The Cross-Industry Standard Process for Data Mining (CRISP-DM) methodology was applied using samples of 89 patients from the *Unidad de Diagnóstico en Patología,* covering all four HER2 expression levels. Deep learning techniques were employed, leveraging neural networks and vision transformer models through transfer learning. Additionally, a usability evaluation was conducted on the final version of the software.

**Results.:**

The ViT-B/16 model achieved a classification accuracy of 90,65%, while the tool was evaluated with an acceptable level of satisfaction in its clinical application.

**Conclusion.:**

Artificial intelligence demonstrated high accuracy and consistency in HER2 classification, reducing diagnostic variability and improving objectivity. However, further optimization of processing efficiency is required for broader applicability.

El cáncer de mama es una enfermedad en la que las células malignas se desarrollan en el tejido mamario, afectando la salud y la calidad de vida de las pacientes. Su tratamiento varía según el tipo y la etapa del cáncer, e incluye cirugía, radioterapia, quimioterapia, terapia hormonal y terapia dirigida ^1^. Para diagnosticar el cáncer y clasificar su agresividad, se emplean técnicas como la inmunohistoquímica que detecta la expresión de proteínas en el tejido analizado.

Una de las proteínas clave es el receptor del factor de crecimiento epidérmico humano de tipo 2 (*Human Epidermal Receptor-2,* HER2) ^2^. El HER2 regula el crecimiento y la división celular, pero su sobreexpresión puede provocar proliferación descontrolada y tumores malignos ^3^. Según el *College of American Pathologists,* todos los cánceres de mama deben analizarse para evaluar la expresión del HER2 ^4^, cuyos resultados de inmunohistoquímica (IHQ) se interpretan así ^5^:

- IHQ 0+: el HER2 es negativo cuando no hay tinción o cuando la tinción de membrana es incompleta, tenue o apenas perceptible en menos del 10 % de las células tumorales.
- IHQ 1+: indica un HER2 negativo cuando la tinción de membrana es incompleta, tenue o apenas perceptible en más del 10 % de las células tumorales.
- IHQ 2+: se considera un HER2 equívoco cuando la tinción de membrana es completa y de intensidad débil a moderada en más del 10 % de las células tumorales o cuando se tiñen con intensidad en menos del 10 %.
- IHQ 3+: indica un HER2 positivo cuando la tinción de membrana es completa e intensa en más del 10 % de las células tumorales.

En el 2020, se reportaron 2,3 millones de nuevos casos de cáncer de mama en el mundo, lo que representa el 11,7 % de todos los cánceres, con 685.000 muertes (6,9 % del total) ^6^. En Colombia, el Departamento Administrativo Nacional de Estadística (DANE) registró 3.671 casos en mujeres y 77 muertes en el departamento del Cauca, de las cuales 31 ocurrieron en Popayán ^7^.

Dada la alta incidencia del cáncer de mama y el impacto clínico de una correcta identificación del biomarcador HER2, es de gran trascendencia optimizar los procesos diagnósticos para garantizar decisiones terapéuticas precisas. La evaluación del HER2 mediante inmunohistoquímica continúa siendo un procedimiento con cierto grado de subjetividad, especialmente en los niveles intermedios 1+ y 2+ 8, lo que puede conducir a errores en la clasificación y, por ende, en la elección del tratamiento dirigido. En este contexto, el desarrollo de sistemas automáticos basados en la inteligencia artificial representa una oportunidad significativa para fortalecer la reproducibilidad, reducir la variabilidad intraobservador e interobservador, y apoyar el diagnóstico histopatológico.

En diversos estudios se han explorado métodos computacionales para la clasificación del HER2, con resultados prometedores. Algoritmos como LMBNet han alcanzado una exactitud del 96,92 % en la clasificación del HER2 ^8^. Los métodos basados en el aprendizaje profundo y *Monte Carlo Dropout,* lograron el 89 % de exactitud en la clasificación del tejido mediante el HER2 ^2^. Un modelo explicable de aprendizaje automático obtuvo una precisión del 88 %, exactitud del 89 % y recuperación del 43 % evaluando el impacto de datos FISH ^9^.

Por otro lado, el método HER2-ResNet, inspirado en redes convolucionales y residuales, alcanzó una exactitud del 93 % ^10^. Además, el uso de modelos de redes generativas *(Generative Adversarial Network,* GAN) ha permitido la generación de imágenes sintéticas para mejorar el entrenamiento, logrando hasta el 94,2 % de exactitud con InceptionResNetV2 ^11^. Por último, se tiene un estudio sobre el muestreo piramidal aplicado a DenseNet-201, el cual obtuvo una exactitud del 84,7 % en la clasificación del HER2 en imágenes de núcleos de tejido mamario ^12^. A pesar de estos avances, persisten desafíos técnicos y clínicos, incluyendo la interpretabilidad de los modelos. Por ello, estas herramientas deben ser validadas por patólogos expertos para su aplicación efectiva.

El objetivo de este estudio fue desarrollar un sistema para la clasificación automática de la sobreexpresión de HER2 en imágenes histológicas de tejido mamario canceroso, mediante el uso de técnicas avanzadas de inteligencia artificial (figura 1). Considerando la variabilidad presente en la evaluación del HER2, se propone un enfoque que integra modelos de aprendizaje profundo, orientado a mejorar la precisión diagnóstica, reducir la subjetividad del observador en el proceso de interpretación y proporcionar una herramienta de apoyo clínico que contribuya a la toma de decisiones en el manejo del cáncer de mama.

**Figura 1:**
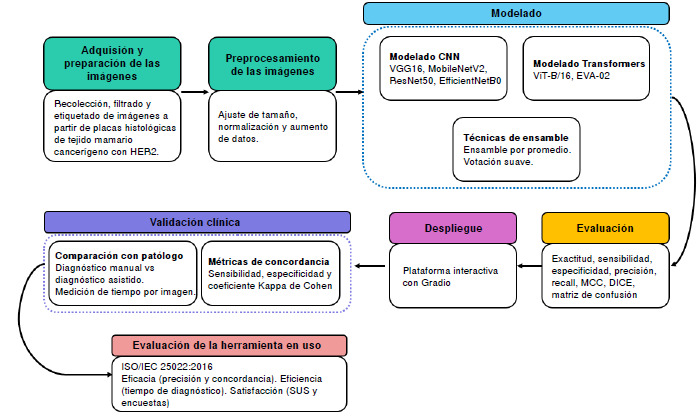
Diagrama de flujo del sistema propuesto para la clasificación de imágenes de cáncer de mama con sobreexpresión de HER2

## Materiales y métodos

En la implementación del sistema propuesto, se usaron dos técnicas de inteligencia artificial: las redes neuronales profundas y los modelos basados en *Transformers.* Ambas técnicas se usaron en el entrenamiento, siguiendo la metodología de transferencia de aprendizaje, y utilizando un conjunto propio de datos construido a partir de imágenes histológicas de tejido mamario canceroso.

### Adquisición de imágenes

La base de datos se construyó en el Laboratorio de Inmunología y Biología Molecular de la Universidad del Cauca. Se adquirieron 58.826 imágenes microscópicas de tejido mamario canceroso procesado con inmunohistoquímica, utilizando un microscopio Leica DM500™ con cámara ICC50W a un aumento de 40X. Tras una selección basada en la calidad histológica y la ausencia de artefactos, 28.507 imágenes se consideraron aptas para el análisis.

### Criterios de inclusión y exclusión

*Criterios de inclusión:* imágenes con tinción adecuada, bien enfocadas y representación completa de las estructuras celulares.

*Criterios de exclusión:* imágenes con desenfoque, sobreexposición, tinción deficiente o presencia de artefactos histológicos.

### Consideraciones éticas

La información se recolectó siguiendo las normas éticas, garantizando la privacidad y la confidencialidad de los datos de los pacientes mediante el anonimato de las muestras. Además, se veló por el principio de no maleficencia, asegurándose de que el proceso de recolección de muestras no causara ningún daño a los pacientes. La investigación se diseñó, también, con el objetivo de beneficiar a la salud pública, aportando una herramienta al avance en el diagnóstico del cáncer de mama.

El protocolo de investigación fue aprobado por el Comité de Ética en Investigación de la Universidad del Cauca. Para garantizar la confidencialidad y el cumplimiento de las consideraciones éticas, se firmó una carta de solicitud que aseguraba el acceso a muestras de una fuente autorizada sin revelar información sensible de los pacientes.

### Distribución de datos

Los datos se organizaron en las cuatro categorías de HER2 (0+, 1+, 2+, 3+) y se dividieron en conjuntos de entrenamiento (70 %), validación (15 %) y prueba (15 %).

El tamaño de la muestra se determinó considerando la disponibilidad de muestras válidas por la categoría de HER2, la necesidad de garantizar el balance y la representatividad entre cuatro categorías. La cantidad final de 28.507 imágenes ofreció un volumen adecuado para evitar el sobreajuste y garantizar la estabilidad estadística en la fase de validación.

### Modelado

*Enfoque.* Para construir los modelos, se usaron dos técnicas: una con las redes neuronales profundas y otra con los *Transformers.*

*Redes neuronales convolucionales (Convolutional Neural Network,* CNN). Son modelos de aprendizaje profundo diseñados para procesar imágenes mediante la aplicación de filtros o núcleos que extraen las características relevantes, lo cual permite aprender patrones repetitivos en toda la imagen. Tras cada operación de convolución, la ventana se mueve y se capturan las características en los mapas de características. Estos mapas recogen el campo receptivo local de la imagen, utilizando pesos y sesgos compartidos ^13^.

*Transformers.* Estos modelos usan una arquitectura de redes neuronales basada en el mecanismo de *self-attention,* lo que les permite asignar importancia a diferentes elementos de una secuencia sin importar su posición. Esta capacidad los hace eficientes para procesar secuencias largas, superando las limitaciones de las redes neuronales recurrentes y las redes neuronales convolucionales ^14^. Su estructura principal incluye un codificador para procesar la entrada y un decodificador para generar la salida. Los *transformers* han revolucionado el procesamiento del lenguaje natural y han ampliado su aplicación a áreas como la visión por computadora ^14^.

Un transformador de visión *(Vision Transformer,* ViT) adapta la arquitectura del modelo *Transformer* para tareas de visión por computadora, divide las imágenes en parches de tamaño fijo que las trata como "palabras" en una secuencia, procesándolas con capas de atención para capturar relaciones globales entre regiones. A diferencia de las redes neuronales convolucionales, el transformador de visión no utiliza convoluciones, lo que le permite superar limitaciones espaciales y destacarse en la clasificación de imágenes, especialmente con grandes volúmenes de datos de entrenamiento ^15^.

### Construcción del modelo

*Definición de parámetros e hiperparámetros.* El entrenamiento de redes neuronales profundas requiere establecer hiperparámetros clave, como el número de capas, las neuronas por capa y las funciones de activación, que permanecen constantes durante el proceso. También, se elige una función de pérdida, un optimizador y la cantidad de épocas para mejorar la precisión del modelo. En el caso de los transformadores de visión, se ajustan parámetros específicos, como el número de capas de transformadores, el tamaño de las representaciones vectoriales *(embeddings),* la cantidad de encabezados *(heads)* de la atención, el tamaño de imagen de entrada y el método de división en parches ^16^^,^^17^. Además, se optimizan la tasa de aprendizaje y el tamaño del lote para mejorar el rendimiento. Los modelos aquí descritos se entrenaron siguiendo la metodología de transferencia de aprendizaje.

*Aprendizaje por transferencia.* Este reutiliza un modelo previamente entrenado en un conjunto de datos grande y lo ajusta para una tarea específica, entrenándolo con menos datos. En las redes neuronales profundas, se congelan capas del modelo original y se ajustan solo las finales, con lo cual se acelera el entrenamiento y se mejora el rendimiento ^18^. El afinamiento de un transformador de visión consiste en cargar los pesos preentrenados, reemplazar la capa de clasificación según el número de clases del nuevo conjunto de datos y, en algunos casos, congelar las capas inferiores para conservar las características generales.

*Técnicas de regularización.* Para evitar el sobreajuste y mejorar la generalización del modelo, se utilizaron técnicas de regularización -entre ellas, *Early Stopping* que detiene el entrenamiento cuando el rendimiento en validación empeora- y el aumento de datos que genera variaciones en las imágenes para fortalecer el modelo, especialmente en el dominio médico.

Debido a su eficiencia y capacidad para manejar la clasificación de las imágenes, se consideraron cuatro redes neuronales profundas: VGG16 ^19^, MobileNetV2 ^20^, ResNet50 ^19^ y EfficientNetB0 ^21^; también, se utilizaron dos modelos de *Vision Transformer:* EVA02 ^22^ y ViT-B/16 ^15^.

### *Arquitectura de modelos de* redes neuronales convolucionales

*VGG16.* La red VGG16 se entrenó en la base de datos ImageNet ^23^. Consta de 16 capas de convolución y tiene un campo receptivo pequeño de 3 * 3. Tiene una capa de agrupación máxima de tamaño 2 * 2 y tiene un total de cinco capas de este tipo. Hay tres capas completamente conectadas después de la última capa de agrupación máxima. Enseguida, se presentan tres capas completamente conectadas. Se utiliza el clasificador *softmax* como capa final. La activación ReLu se usa en todas las capas ocultas ^19^.

*MobileNetV2.* Esta red neuronal ha utilizado convoluciones ligeras en profundidad para filtrar características. Comienza con una convolución estándar inicial (3 * 3, *stride* 2) seguida de bloques *Inverted Residual* con convoluciones *depthwise* separables, que incluyen una etapa de expansión (conv 1 * 1), convolución *depthwise* (3 * 3) y proyección (conv 1 * 1), con conexiones residuales, según corresponda. Estos bloques están distribuidos estratégicamente con diferentes configuraciones de expansión, filtros y *strides.* Finaliza con una convolución 1 * 1 para expandir canales, una capa de *pooling* global promedio y una capa completamente conectada con activación *softmax* para la clasificación ^20^.

*ResNet-50.* Esta es una forma abreviada de redes residuales que tiene 50 capas. Es comparable con la VGG16, excepto que ResNet-50 tiene una capacidad adicional de mapeo de identidad. Esta red neuronal predice el delta que se requiere para alcanzar la predicción final de una capa a la siguiente. También, reduce el problema del gradiente evanescente, al permitir que este camino alternativo de atajo fluya a través del gradiente. El mapeo de identidad utilizado en ResNet permite que el modelo omita una capa de peso de las redes neuronales convolucionales si la capa actual no es necesaria. Esto ayuda a evitar el problema de sobreajuste al conjunto de entrenamiento ^19^.

*EfficientNetBO.* Esta red es el modelo base de la familia, diseñado desde cero mediante un proceso de búsqueda automática de arquitectura *(NeuralArchitecture Search,* NAS). Su arquitectura se construye utilizando bloques MBConv *(Mobile Inverted Bottleneck Convolution),* combinados con técnicas de atención de canales *(Squeeze-and-Excitation,* SE) y un esquema de escalado compuesto para optimizar el rendimiento. Utiliza un método de escalado compuesto para ajustar simultáneamente la profundidad, la anchura y la resolución de la red, logrando un balance óptimo entre precisión y eficiencia. Con solo 5,3 millones de parámetros, emplea la función de activación *swish* y técnicas de regularización como *dropout*^21^.

### Arquitectura de modelos Transformer

*EVA-02.* El modelo *Enhanced Vision Transformer Architecture* (EVA-02) es una versión optimizada del transformador de visión, el cual mejora la eficiencia en las tareas de visión por computadora. Ajusta la división de imágenes en parches para capturar mejor las características locales, modifica los bloques de atención para una asignación más eficiente de los recursos y el procesamiento rápido de las imágenes de alta resolución. EVA-02 es útil en la clasificación y la segmentación de imágenes médicas, y en otras aplicaciones que requieren gran calidad y resolución ^22^.

*ViT-B/16.* El modelo ViT-B/16 es una variante del transformador de visión, donde B indica la configuración "base" adaptada de *Bidirectional Encoder Representations from Transformers* (BERT) y "/16" significa que la imagen de entrada se divide en parches de 16 x 16 píxeles. Cuenta con 12 capas, un tamaño oculto de 768, un "perceptrón" de múltiples capas *(Multi-Layer Perceptron,* MLP) de un tamaño de 3.072, 12 encabezados de atención y 86 millones de parámetros. Además, el largo de la secuencia del transformador de visión es inversamente proporcional al cuadrado del tamaño del parche, lo que implica que los modelos con parches más pequeños requieren mayor capacidad computacional ^15^.

### Técnicas de ensamble de modelos

Para mejorar la precisión y la solidez del modelo, se implementaron dos técnicas de ensamblado: ensamble por promedio y clasificador por votación.

- *Ensamble por promedio (average ensemble).* Permite combinar múltiples modelos base mediante un promedio ponderado, reduciendo la tasa de error y la varianza, lo que mejora la capacidad de generalización del sistema ^24^.
- *Clasificador por votación* ( *VotingClassifier).* Combina las predicciones de varios modelos para obtener un mejor desempeño. Puede ser votación dura, en la cual la clase con más votos es la predicción final, o votación blanda, que pondera las probabilidades de cada modelo, lo que resulta útil en caso de distribuciones desequilibradas. Este método es ampliamente utilizado en visión por computadora y bioinformática ^25^.

### Validación y evaluación

La evaluación del modelo se llevó a cabo en dos sentidos: el primero, la evaluación del desempeño del modelo, y, el segundo, el grado de aceptación por parte de los patólogos. Para ello, se implementó una interfaz optimizada con Gradio, una librería de código abierto para Python que facilita la creación de interfaces web interactivas en el aprendizaje automático y en la ciencia de datos ^26^. La plataforma permite cargar imágenes para su análisis y muestra la clasificación correspondiente en la misma sección de visualización. Este proceso puede repetirse para analizar nuevas imágenes de manera sencilla e intuitiva.

### Evaluación del desempeño del modelo

Se evaluó el rendimiento del modelo utilizando datos de prueba no vistos previamente por el modelo, garantizando la reiteración externa del modelo. Se emplearon mediciones clave para establecer su capacidad predictiva, como se lista n a continuación:

*Matriz de confusión:* resume el desempeño del modelo en términos de verdaderos positivos *(true positive,* tp), falsos positivos *(false positive,* fp), verdaderos negativos *(true negative,* tn) y falsos negativos *(false negative,* fn).

*Exactitud:* representa la proporción de predicciones correctas sobre el total de muestras evaluadas.




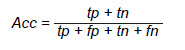




*Sensibilidad y especificidad:* miden la capacidad del modelo para identificar correctamente los casos positivos y negativos, respectivamente.




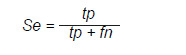







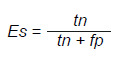




*Precisión y recordación (recall):* evalúan la proporción de casos positivos correctamente identificados y la capacidad de recuperar los casos positivos reales.




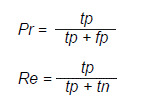




*Coeficiente de correlación de Matthews* (MCC): proporciona una evaluación más equilibrada del rendimiento del modelo, y es útil en conjuntos de datos desequilibrados.




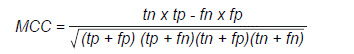




*Coeficiente de similitud de Dice:* aplicado en la segmentación de imágenes médicas, mide la superposición entre predicciones y etiquetas reales.




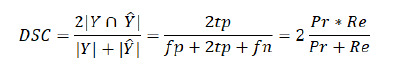




### Evaluación en el entorno clínico

Se estudió la concordancia de la clasificación del nivel del HER2 en tejido mamario canceroso, con la participación de un patólogo. Los resultados se analizaron mediante la sensibilidad, la especificidad y el coeficiente kappa de Cohen, el cual cuantifica la concordancia entre evaluadores, o entre el modelo y los expertos clínicos. Un valor cercano a uno indica gran concordancia, mientras que los valores bajos sugieren que el acuerdo es similar al azar.

Se compararon dos métodos:

- *Evaluación convencional:* clasificación visual basada en criterios clínicos.
- *Evaluación asistida por software:* predicción automatizada con revisión del especialista.

### Evaluación de la herramienta

En el proceso de evaluación de la herramienta para clasificar los niveles de HER2, se siguió la norma ISO/IEC 25022:2016, en la que se establece un conjunto de mediciones para evaluar la calidad del uso de un sistema o *software* desde la perspectiva del usuario final ^27^. El estándar propone cinco etapas en el proceso de evaluación: definición, diseño, planificación, ejecución y conclusión. El objetivo principal es garantizar que el sistema cumpla con los criterios de calidad en términos de eficacia, eficiencia y satisfacción.

### Definición de la evaluación

Se establecieron los objetivos para evaluar la calidad del desempeño de la herramienta en el diagnóstico del nivel de HER2 en tejido mamario canceroso, priorizando tres características clave:

- Eficacia (50 %): precisión y concordancia con el criterio de los patólogos.
- Eficiencia (30 %): tiempo requerido en comparación con el método tradicional.
- Satisfacción (20 %): facilidad de uso e integración en el flujo de trabajo.

### Diseño de la evaluación

En esta se definieron las mediciones, métodos y criterios de éxito para evaluar cada subcaracterística. Cada subcaracterística fue ponderada de acuerdo con su impacto en la herramienta.

En la evaluación de la herramienta, se establecieron tres características principales, cada una con un nivel de importancia y ponderación específicos. La eficacia se consideró la característica más relevante, con una ponderación del 50 %; se evaluó mediante el análisis de la concordancia entre los resultados del modelo y los del criterio del patólogo. La eficiencia obtuvo una ponderación del 30 %, determinada a partir del tiempo medio requerido para el diagnóstico con uso del *software* y sin él. Finalmente, la satisfacción se asignó con una ponderación del 20 %, evaluada mediante las encuestas sobre la escala de facilidad de uso del sistema *(System Usability Scale,* SUS) y la retroalimentación proporcionada por los patólogos participantes.

### Especificación de las mediciones

Se definieron las mediciones correspondientes para evaluar cada subcaracterística del sistema, junto con su propósito y método de medición.

Para evaluar la concordancia, se emplearon parámetros como la sensibilidad, la especificidad y el índice kappa, con el fin de medir la precisión y la confiabilidad del diagnóstico mediante el *software,* en comparación con los diagnósticos realizados por los patólogos, así como el nivel de concordancia en la clasificación del HER2. El procedimiento consistió en comparar los resultados del *software* con el diagnóstico de referencia de los especialistas y hacer las respectivas mediciones.

En cuanto al tiempo medio para el diagnóstico, con *software* y sin él, se consideraron indicadores como el tiempo promedio por imagen, y la diferencia de tiempo entre el método tradicional y el asistido por el *software.* Esta medición tuvo como propósito determinar si la herramienta mejorab a la eficiencia del proceso diagnóstico sin afectar la exactitud, mediante la medición del tiempo que tarda un patólogo en analizar cada imagen bajo ambas condiciones.

Finalmente, para la satisfacción, se hicieron encuestas sobre la escala de facilidad de uso del sistema y se recopiló la retroalimentación de los patólogos, con el objetivo de evaluar la facilidad de uso, la aceptación y la integración del *software* en el flujo de trabajo clínico. Este proceso incluyó el uso de las encuestas después de utilizar la herramienta, y el análisis de las tendencias y del nivel general de satisfacción reportado por los participantes.

### Definición de la puntuación de la calidad del desempeño

Para interpretar los resultados obtenidos, se tuvieron en cuenta los niveles según la puntuación y los grados de satisfacción. Para la evaluación de la satisfacción, se estableció una escala de medición basada en rangos porcentuales que determinan el grado de cumplimiento de los requisitos del *software.* Los valores entre 79,1 y 100,0 indican un desempeño muy satisfactorio, correspondiente a un cumplimiento pleno de los requisitos.

Los valores entre 49,1 y 79,0 reflejan un resultado satisfactorio o aceptable. Cuando el puntaje se ubica entre 19,1 y 49,0, se considera no satisfactorio, aunque mínimamente aceptable. Finalmente, los valores entre 0,0 y 19,0 representan un desempeño inaceptable e indican que el sistema no cumple con los estándares esperados.

### Planificación de la evaluación

En esta etapa, se decidió cómo se llevaría a cabo la evaluación del sistema, incluyendo las tareas a cargo de los usuarios y los instrumentos de medición utilizados. Las principales actividades fueron las siguientes:

*Definición de tareas para medir la eficacia y la eficiencia.* Se establecieron las siguientes tareas específicas a cargo del patólogo que evaluaría la herramienta:

- Acceder al *software* y cargar las imágenes de tejido mamario.
- Clasificar manualmente de las imágenes sin el *software* de apoyo.
- Llevar a cabo la clasificación asistida con el *software.*
- Comparar el diagnóstico manual con el diagnóstico del *software* y hacer ajustes si se considerare necesario.
- Registrar el tiempo empleado en cada caso (con *software* y sin él),

y evaluar el desempeño según el grado de eficiencia temporal. Para ello, se establecieron rangos que clasifican el rendimiento del sistema en función del tiempo promedio requerido durante el diagnóstico. Los valores entre 75,1 y 100,0 corresponden a un grado muy eficiente, y evidencian una reducción significativa del tiempo. Los valores entre 50,1 y 75,0 corresponden a una eficiencia moderada, con una mejora observable pero no sobresaliente. Los puntajes entre 25,1 y 50,0 se interpretan como neutros e indican una diferencia mínima entre los métodos comparados, mientras que los valores entre 0,0 y 25,0 indican ineficiencia, al no presentar una mejora significativa o, incluso, mostrar un aumento en el tiempo requerido.

- Exportar los resultados en el formato establecido para el análisis de concordancia.

*Diseño de la encuesta de facilidad de uso.* Para evaluar la satisfacción y la comodidad con el desempeño, se decidió utilizar la escala de facilidad de uso del sistema ^28^, una herramienta estandarizada que proporciona una evaluación global de la percepción de la facilidad de uso. La encuesta consta de diez preguntas que el participante califica en una escala de 1 (totalmente en desacuerdo) a 5 (totalmente de acuerdo).

*Diseño de la encuesta de satisfacción.* Para evaluar la satisfacción y la percepción que tienen los patólogos del *software,* se diseñó una encuesta enfocada en la funcionalidad de la herramienta y su utilidad en la práctica clínica. La encuesta consta de ocho preguntas, clasificadas en tres categorías: satisfacción general, percepción sobre la facilidad de uso y percepción sobre la funcionalidad.

Satisfacción general

- ¿Está satisfecho con el apoyo del *software* en la clasificación de HER2?
- ¿El *software* complementa bien su experiencia y conocimiento como patólogo?
- ¿Seguiría usando esta herramienta en futuros casos clínicos?

Percepción sobre la facilidad de uso

- ¿El proceso de carga y análisis de imágenes es sencillo?
- ¿Las herramientas de asistencia son intuitivas?
- ¿El *software* proporciona información clara y útil para la toma de decisiones?

Percepción de funcionalidad

- ¿La clasificación realizada por el *software* es confiable?
- ¿El *software* facilita el análisis de muestras y mejora la eficiencia del diagnóstico?

*Ejecución de la evaluación.* Se implementaron las actividades planificadas para evaluar el *software,* incluyendo la selección del patólogo según su experiencia y la preparación del entorno de pruebas. El patólogo hizo la clasificación manual y asistida, y se registraron las mediciones clave: sensibilidad, especificidad, concordancia kappa y tiempo de diagnóstico. También, se hicieron encuestas sobre la facilidad del uso y la satisfacción, y se recopilaron los datos para su análisis.

*Conclusión de la evaluación.* Se consolidaron los datos obtenidos, se verificó su integridad, se compararon los diagnósticos manuales y asistidos para evaluar la concordancia. Se analizó la puntuación del desempeño del *software* en términos de eficacia, eficiencia y satisfacción.

## Resultados

### Evaluación del desempeño de los modelos

*Modelos de redes neuronales convolucionales.* Se realizó el entrenamiento de los cuatro modelos de redes neuronales convolucionales: VGG16, MobileNetV2, ResNet-50 y EfficientNetB0, mediante la transferencia de aprendizaje y usando el conjunto de las imágenes seleccionadas.

**Figura 2:**
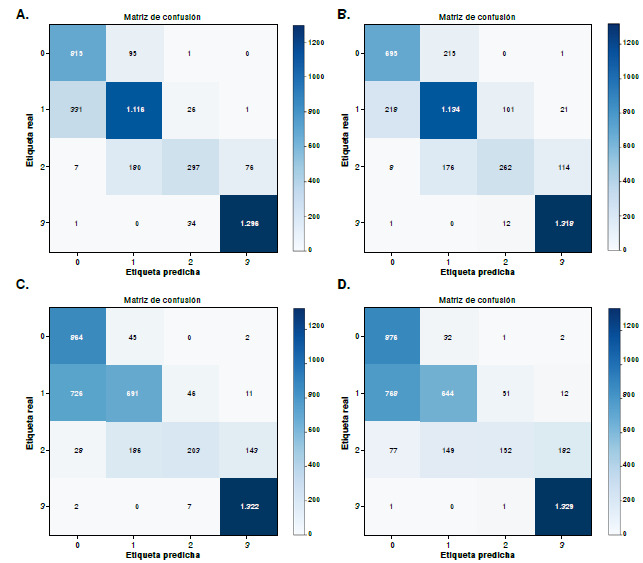
Resultados de las matrices de confusión para los modelos de redes neuronales convolucionales. **A.** Modelo VGG16. **B.** Modelo MobileNetV2. **C.** Modelo ResNet-50. **D.** Modelo EfficientNetB0

Los resultados de exactitud evidenciaron que los modelos que mejor clasificaron las clases 1+ y 2+, fueron VGG16 con el 82,41 % y MobileNetV2 con el 79,72 %, mientras que los que mejor clasificaron las clases 0+ y 3+, fueron ResNet-50 con el 72,03 % y EfficientNetB0 con el 70,16 % (cuadro 1 y figura 2). Teniendo esto en cuenta, se realizó un ensamble mediante promedio y votación blanda con la combinación entre estos modelos (cuadro 2). El ensamble mediante votación blanda de los modelos VGG16 y EfficientNetB0, obtuvo el mejor desempeño en cuanto la exactitud, con el 82,23 %.

**Cuadro 1:** Resultados de las medidas de evaluación del desempeño de los modelos CNN de las redes neuronales convolucionales

Medida	Clase	VGG16	MobileNetV2	ResNet50 EfficientNetB0	
DICE	0	78,93	75,83	68,27	66,54
	1	77,91	75,63	57,68	55,96
	2	64,71	56,04	49,75	39,74
	3	95,86	94,65	94,13	93,07
DICE AVG Sensibilidad		79,35	75,54	67,46	63,83
	0	89,46	76,29	94,84	96,16
	1	75,41	76,93	46,88	43,62
	2	53,04	46,79	36,25	27,14
	3	97,37	99,02	99,32	99,85
Especificidad	0	89,93	93,25	77,53	74,86
	1	90,19	86,05	91,76	93,54
	2	98,36	96,96	98,57	98,57
	3	97,39	95,38	94,70	93,34
Precisión	0	70,62	75,38	53,33	50,87
	1	80,23	74,36	74,95	78,03
	2	82,96	69,87	79,30	74,15
	3	94,39	90,65	89,45	87,15
F1-Score	0	78,93	75,83	68,27	66,54
	1	77,91	75,63	57,68	55,96
	2	64,71	56,04	49,75	39,74
	3	95,86	94,65	94,13	93,07
MCC		75,87	71,74	63,89	62,08
Exactitud		82,41	79,72	72,03	70,16

**Cuadro 2:** Resultado de las técnicas de ensamble para los modelos de las redes neuronales convolucionales

Ensamble mediante promedio		
Modelo 1 (50 %)	Modelo 2 (50 %)	Exactitud
VGG16	ResNet-50	80,03
	EfficientNetB0	81,10
MobileNetV2	ResNet-50	78,13
	EfficientNetB0	79,68
Ensamble mediante votación blanda		
Modelo 1 (70 %)	Modelo 2 (30 %)	Exactitud
VGG16	ResNet-50	81,48
	EfficientNetB0	82,23
MobileNetV2	ResNet-50	80,17
	EfficientNetB0	80,50

**Figura 3:**
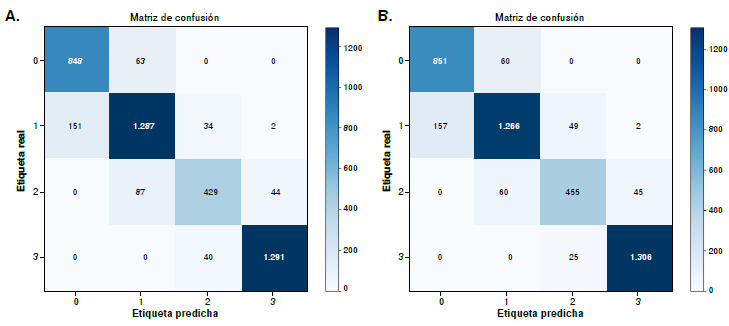
Resultados de las matrices de confusión para modelos *Transformer.* **A.** Modelo EVA02.; **B.** Modelo ViTB16

*Modelos de transformadores de visión.* Se emplearon dos modelos basados en la arquitectura de los transformadores de visión: EVA-02 y ViT-B/16. Ambos fueron entrenados para clasificar las cuatro clases correspondientes a los diferentes grados de sobreexpresión de HER2; los resultados sobre exactitud correspondieron al 90,15 y al 90,69 %, respectivamente (cuadro 3 y figura 3).

**Cuadro 3:** Resultados de las medidas de evaluación del desempeño de los modelos *Vision Transformer*

Medida	Clase	EVA02	ViT-B/16
DICE	0	88,80	88,69
	1	88,42	88,53
	2	80,71	83,56
	3	96,78	97,32
DICE AVG Sensibilidad		88,68	89,53
	0	93,08	93,41
	1	87,31	85,89
	2	76,61	81,25
	3	96,99	98,12
Especificidad	0	95,51	95,33
	1	94,65	95,72
	2	98,01	98,01
	3	98,44	98,40
Precisión	0	84,88	84,42
	1	89,56	91,34
	2	85,29	86,01
	3	96,56	96,53
F1-Score	0	88,80	88,69
	1	88,42	88,53
	2	80,71	83,56
	3	96,78	97,32
MCC		86,40	87,19
Exactitud		90,15	90,69

**Figura 4:**
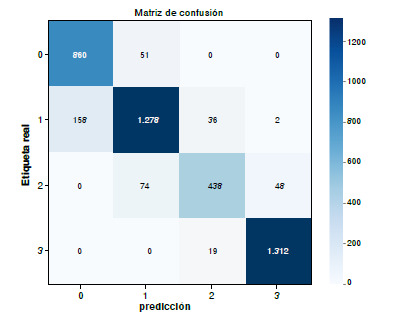
Resultado de la matriz de confusión para el ensamble mediante votación blanda de los modelos ViT-B/16 y VGG16

Se hizo un ensamble por votación blanda, combinando los modelos *Vision Transformer* con el mejor modelo de redes neuronales convolucionales, VGG16 (cuadro 4); además, se calculó la matriz de confusión del mejor modelo (figura 4). Este método fue seleccionado porque permite asignar mayor peso a un modelo, en este caso a los *Vision Transformers,* debido a su alto rendimiento.

**Cuadro 4:** Resultados de la técnica de ensamble mediante votación blanda para *transformers*

Modelo 1 (70 %)	Modelo 2 (30 %)	Exactitud
EVA02	VGG16	74,72
ViT-B/16	VGG16	90,93

### Desarrollo de la herramienta para clasificar el HER2

Se desarrolló una plataforma intuitiva con Gradio para facilitar el acceso del usuario final. Esta herramienta admite imágenes en formatos PNG, JPG, JPEG y BMP, aplicando un preprocesamiento para ajustarlas a las condiciones de entrenamiento del modelo ViT-B/16, optimizado para analizar la sobreexpresión del HER2 en el cáncer de mama. Finalmente, el modelo procesa las imágenes y genera la clasificación y predicción correspondientes.

### Evaluación de la concordancia entre el patólogo y la herramienta de clasificación del HER2

Para evaluar la concordancia entre el patólogo y la herramienta desarrollada, se empezó analizando 20 imágenes con una distribución relativamente equilibrada en cada nivel de HER2 (cuadro 5). Durante este proceso, también se midió el tiempo promedio de diagnóstico por imagen, obteniéndose un valor de 12 segundos por imagen. Por otro lado, con el segundo método que incluye la asistencia del *software* (cuadro 5) en este caso, el tiempo promedio de diagnóstico se extendió a 14 segundos.

**Figura 5:**
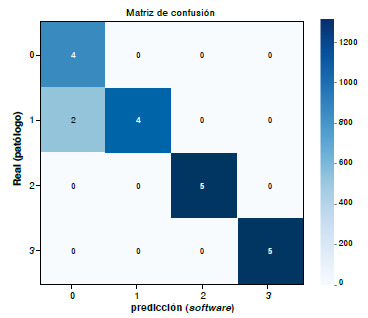
Resultado de la matriz de confusión para la evaluación de concordancia mediante el método de evaluación asistida por *software*

A partir de la matriz de confusión (figura 5), se calcularon la sensibilidad, la especificidad y el índice kappa de Cohen. Los resultados obtenidos con el método asistido muestran una sensibilidad de 0,10 para las clases 0, 2 y 3, y de 0,67 para la clase 1, alcanzando un promedio global de 0,9167 (91,67 %). En términos de especificidad, se registraron valores de 0,875 para la clase 0. y de 0,10 para las clases 1, 2 y 3, con un promedio total de 0,9687 (96,87 %). Finalmente, el coeficiente kappa de Cohen fue de 0,8675 (86,75 %), lo que refleja una gran concordancia entre las predicciones del modelo y las etiquetas reales, y respalda la fiabilidad del sistema como herramienta de apoyo diagnóstico.

**Cuadro 5:** Resultados de la clasificación de veinte imágenes de HER2 mediante el método de evaluación convencional y el método de evaluación asistida

Imagen	Evaluación convencional	Evaluación asistida por el software	
	Nivel de HER2	Nivel de HER2	
	Patólogo	Patólogo	*Software*
0	0	0	0
1	1	2	2
2	1	1	0
3	3	3	3
4	1	1	1
5	3	3	3
6	0	0	0
7	2	2	2
8	3	3	3
9	1	1	1
10	1	2	2
11	3	3	3
12	0	0	0
13	2	2	2
14	1	1	1
15	0	1	1
16	3	3	3
17	0	0	0
18	2	2	2
19	1	1	0

### Evaluación de la herramienta

Para la evaluación integral de la herramienta desarrollada, se consideraron las medidas de concordancia y los indicadores de eficiencia y satisfacción, con el fin de obtener una valoración completa de su desempeño. En primer lugar, se calculó el promedio de las tres medidas principales de concordancia, obteniéndose un valor representativo de la eficacia del sistema, que alcanzó el 91,76 %, con una ponderación del 50 %, clasificada como muy satisfactoria, y con una contribución de 45,88 puntos al resultado final.

La eficiencia se evaluó mediante el tiempo de diagnóstico requerido para la clasificación de imágenes, con asistencia del *software* y sin ella. Se observó un incremento promedio de dos segundos al emplear la herramienta (de 12 a 14 segundos por imagen), diferencia considerada mínima. Por ello, se le asignó una puntuación neutral del 50 %, con una ponderación del 30 %, equivalente a 15 puntos, clasificándose como satisfactoria.

En cuanto a la satisfacción del usuario, los resultados se obtuvieron a partir de las encuestas de la escala de facilidad de uso del sistema y de satisfacción, que registraron puntuaciones de 82,5 y 92,5, respectivamente. El promedio de ambas evaluaciones fue del 87,5 %, correspondiente a un nivel muy satisfactorio, con una ponderación del 20 % y una contribución de 17,5 puntos.

Finalmente, al integrar los valores de las tres dimensiones -eficacia, eficiencia y satisfacción- se obtuvo un puntaje global de 78,38, lo que corresponde a una aceptable calidad del desempeño y a un apropiado grado general de satisfacción. Estos resultados reflejan un desempeño adecuado del sistema como herramienta de apoyo diagnóstico en la clasificación automatizada del HER2, al combinar precisión técnica, facilidad de uso clínico y aceptación por parte de los especialistas.

## Discusión

El conjunto de datos recolectado en esta investigación consta de un volumen considerable de imágenes (58.826) para la clasificación de los niveles de HER2, en comparación con otros trabajos como LMBNet ^8^, HER2-ResNet ^10^ y HERGAN ^11^, cuyos conjuntos de datos oscilan entre las 158 y las 5.200 imágenes.

Esta diferencia representa una fortaleza relevante ya que, al ser un conjunto de datos de tamaño considerable, proporciona una base sólida para el entrenamiento de las arquitecturas empleadas, reduce el sobreajuste y mejora la generalización del modelo. Además, al tratarse de imágenes reales y no sintéticas, se incrementa la confianza del patólogo, ya que asegura una mejor concordancia con las observaciones que hace el especialista.

El modelo ViT-B/16 alcanzó una exactitud del 90,69 %, superando el modelo de estimación de incertidumbre (89 %) ^2^, aunque este último se enfoca en cuantificar la incertidumbre mediante el *Monte Carlo dropout* para mejorar la interpretabilidad visual, un aspecto no abordado en el presente estudio. De forma similar, la regresión logística basada en inmunohistoquímicaca, reportó 89 % de exactitud y 93 % al incorporar datos FISH ^9^, priorizando la interpretabilidad sobre el rendimiento, mientras que este trabajo se orienta hacia la validación práctica con patólogos.

Comparado con modelos como LMBNet con el 96,92 % ^8^, HER2-ResNet con el 93 % ^10^ e InceptionResNetV2 con el 94,2 % ^11^, el rendimiento del modelo ViT-B/16 es ligeramente inferior. No obstante, la diferencia puede atribuirse a la variabilidad en los conjuntos de datos utilizados en los estudios, puesto que algunos son privados o contienen imágenes sintéticas, mientras que este estudio desarrolló un conjunto propio de datos, lo cual permitió un mayor control sobre la calidad, la diversidad y la relevancia clínica de las muestras.

Una fortaleza clave del presente trabajo radica en la evaluación integral del modelo, que incluye mediciones de la exactitud, la sensibilidad, la especificidad, el índice de Matthews, el coeficiente de similitud de dados y el índice kappa de Cohen. Este análisis es más amplio que el de otros estudios que suelen centrarse únicamente en la exactitud, la precisión, la recordación y el puntaje F1.

Además, este trabajo introduce un enfoque diferencial al incorporar la evaluación con patólogos mediante encuestas sobre la escala de facilidad de uso del sistema y de satisfacción, conforme a la norma ISO/IEC 25022:2016, lo que permite medir la la calidad del desempeño desde la perspectiva del usuario final. Esta validación clínica y de facilidad de uso es poco común en la literatura científica y refuerza la aplicabilidad práctica del sistema como herramienta de apoyo diagnóstico.

Los modelos de redes neuronales convolucionales alcanzaron un máximo del 82,41% de exactitud, destacándose en la clasificación de los niveles 0+ y 3+, mientras que los niveles 1+ y 2+ presentaron mayor dificultad, atribuida a la similitud entre clases (figura 1). Aunque los modelos como HER2GAN ^11^ obtienen mejores resultados, alcanzando una exactitud del 94,2% con InceptionResNetV2 al usar datos sintéticos, la propuesta de este estudio ofrece una ventaja significativa en términos de confiabilidad clínica al emplear únicamente imágenes reales.

Asimismo, se implementaron dos tipos de ensamble: el ensamble por promedio, en el que a los dos modelos se les asignó el mismo valor de importancia, el 50 % cada uno, alcanzando una exactitud entre el 78 y el 81,10 %, que superó a los modelos individuales ResNet50 y EfficientNetB0, pero no al modelo VGG16. Por otro lado, en la votación blanda, se asignó un mayor peso al modelo 1 del 70 % y un menor peso al modelo 2 del 30 %, ya que MobileNetV2 y VGG16, incluidos en el modelo 1, tuvieron un mejor rendimiento en los niveles del HER2 de interés: 1+ y 2+. Se obtuvieron exactitudes entre el 80 y el 82,23 %, un rendimiento superior al ensamble por promedio, aunque aún por debajo de la red VGG16.

Los modelos EVA-02 y ViT-B/16, entrenados con *EarlyStopping,* alcanzaron exactitudes del 90,15 y el 90,69 %, respectivamente. Ambos mostraron un desempeño destacado en los niveles 0+ y 3+, y los niveles 1+ y 2+ continuaron siendo como los más desafiantes (figura 2). Si bien EVA-02 se ubica ligeramente por debajo de HER2-ResNet con el 93% de exactitud, e InceptionResNetV2 con el 94,2%, ofrece ventajas en interpretabilidad al prescindir de las convoluciones tradicionales.

La combinación de ViT-B/16 con VGG16 mejoró la exactitud global al 90,93 %, aunque con un menor rendimiento en la clase 2+, de especial interés clínico. Este resultado sugiere que los modelos híbridos pueden mejorar la exactitud general.

En general, los resultados de sensibilidad y especificidad superiores al 90 %, junto con un índice kappa mayor de 0,85, confirman la gran fiabilidad del sistema. El estudio aporta un enfoque integral que combina técnicas avanzadas de aprendizaje profundo con una validación clínica y de facilidad de uso, lo cual constituye un aporte significativo al campo de la clasificación automatizada del HER2.

Aunque el valor diagnóstico de la herramienta es evidente, sus aplicaciones podrían extenderse más allá del entorno clínico inmediato. Por ejemplo, podría emplearse como herramienta de apoyo en la formación de los residentes de patología, o como sistema asistido en los laboratorios con menor capacidad diagnóstica, donde facilitaría la detección temprana de los casos sospechosos y estandarizaría los criterios de evaluación.

Si bien los resultados obtenidos son prometedores, se identifican algunas limitaciones del presente estudio. El entrenamiento se realizó sobre una base de datos institucional única, lo cual podría restringir la capacidad de generalización del modelo a otros entornos clínicos. Asimismo, pueden existir sesgos en la selección de imágenes, derivados de las condiciones de captura o de la variabilidad en las tinciones utilizadas.

Se considera necesaria una validación externa multicéntrica que permita confirmar la solidez del modelo en diferentes contextos hospitalarios. Además, el tiempo de respuesta computacional aún puede optimizarse, dado que la eficiencia temporal obtuvo una puntuación del 50 %, lo que refleja la necesidad de mejorar la velocidad de procesamiento del sistema.

Por otro lado, la variabilidad en la clasificación de los niveles 1+ y 2+ continúa siendo un desafío, común en la literatura, debido a la similitud de los patrones visuales. Las futuras investigaciones podrían explorar enfoques basados en la atención con múltiples escalas, el aumento de los datos específicos para las clases intermedias o los modelos multimodales que integren información de tinciones complementarias como FISH.

El uso de la inteligencia artificial, específicamente el modelo *Vision Transformer* ViT-B/16 dentro de la metodología CRISP-DM *(Cross-Industry Standard Process for Data Mining),* permitió automatizar con gran precisión la detección y la cuantificación del HER2 en tejido mamario canceroso, alcanzando el 90,69 % de exactitud. La evaluación del sistema evidenció su eficacia y facilidad de uso, con un nivel de satisfacción aceptable en su aplicación clínica, aunque se identificó que la optimización del tiempo de procesamiento es un aspecto por mejorar. Los resultados reflejan una gran concordancia diagnóstica, reduciendo la subjetividad y mejorando la confiabilidad en la selección de tratamientos personalizados, lo que fortalece su aplicabilidad en la práctica clínica.

Las contribuciones de este trabajo son: la creación de un conjunto de datos de imágenes que documenta tejidos mamarios con los cuatro niveles de expresión del HER2; la implementación de modelos de la inteligencia artificial para la identificación precisa del estado del HER2, con el potencial de asistir a los patólogos en decisiones clínicas, y la adopción de una herramienta por parte de los especialistas que podría reducir la variabilidad a la hora de hacer los diagnósticos, haciendo que la evaluación de las muestras histológicas sea más efectiva y optimizando el proceso para un tratamiento dirigido.

Además, este trabajo sienta las bases para seguir usando la inteligencia artificial en el futuro en la ampliación del análisis de biomarcadores importantes en el cáncer de mama -como Ki-67- lo cual permitirá desarrollar modelos más completos y personalizados para la clasificación del cáncer de mama.
